# Interactions and Potential Implications of *Plasmodium falciparum*-Hookworm Coinfection in Different Age Groups in South-Central Côte d'Ivoire

**DOI:** 10.1371/journal.pntd.0001889

**Published:** 2012-11-01

**Authors:** Aurélie A. Righetti, Dominik Glinz, Lukas G. Adiossan, Ahou-Yah G. Koua, Sébastien Niamké, Richard F. Hurrell, Rita Wegmüller, Eliézer K. N'Goran, Jürg Utzinger

**Affiliations:** 1 Department of Epidemiology and Public Health, Swiss Tropical and Public Health Institute, Basel, Switzerland; 2 University of Basel, Basel, Switzerland; 3 Laboratory of Human Nutrition, Institute of Food, Nutrition, and Health, ETH Zurich, Zurich, Switzerland; 4 Hôpital Général de Taabo, Taabo Cité, Côte d'Ivoire; 5 Unité de Formation et de Recherche Biosciences, Université de Cocody, Abidjan, Côte d'Ivoire; 6 Centre Suisse de Recherches Scientifiques en Côte d'Ivoire, Abidjan, Côte d'Ivoire; DVBNTD/CWRU/Emory University, Kenya

## Abstract

**Background:**

Given the widespread distribution of *Plasmodium* and helminth infections, and similarities of ecological requirements for disease transmission, coinfection is a common phenomenon in sub-Saharan Africa and elsewhere in the tropics. Interactions of *Plasmodium falciparum* and soil-transmitted helminths, including immunological responses and clinical outcomes of the host, need further scientific inquiry. Understanding the complex interactions between these parasitic infections is of public health relevance considering that control measures targeting malaria and helminthiases are going to scale.

**Methodology:**

A cross-sectional survey was carried out in April 2010 in infants, young school-aged children, and young non-pregnant women in south-central Côte d'Ivoire. Stool, urine, and blood samples were collected and subjected to standardized, quality-controlled methods. Soil-transmitted helminth infections were identified and quantified in stool. Finger-prick blood samples were used to determine *Plasmodium* spp. infection, parasitemia, and hemoglobin concentrations. Iron, vitamin A, riboflavin, and inflammation status were measured in venous blood samples.

**Principal Findings:**

Multivariate regression analysis revealed specific association between infection and demographic, socioeconomic, host inflammatory and nutritional factors. Non-pregnant women infected with *P. falciparum* had significantly lower odds of hookworm infection, whilst a significant positive association was found between both parasitic infections in 6- to 8-year-old children. Coinfected children had lower odds of anemia and iron deficiency than their counterparts infected with *P. falciparum* alone.

**Conclusions/Significance:**

Our findings suggest that interaction between *P. falciparum* and light-intensity hookworm infections vary with age and, in school-aged children, may benefit the host through preventing iron deficiency anemia. This observation warrants additional investigation to elucidate the mechanisms and consequences of coinfections, as this information could have important implications when implementing integrated control measures against malaria and helminthiases.

## Author Summary

In sub-Saharan Africa, parasitic worms (helminths) are among the most common chronic infections, malaria among the most deadly, and coinfection is the norm rather than the exception. Infections with hookworm and *Plasmodium* can decrease the level of hemoglobin and are therefore associated with anemia. Previous studies have investigated the consequences of coinfection in different age groups and settings, but results are conflicting. Indeed, there is no consensus about detrimental or beneficial effects of a coinfection for the host. We designed a cross-sectional study to determine risk factors for anemia and investigated interactions and discuss potential implications of *P. falciparum* and hookworm coinfection in three groups of people. Overall, 324 individuals were diagnosed for helminths and *Plasmodium* infections, anemia, subclinical inflammation, and micronutrient deficiencies, and household’s socioeconomic status was determined based on an asset-index. We found significant associations between hookworm and *P. falciparum* infections, depending on the age group. Interestingly, 6- to 8-year-old children harboring a coinfection showed significantly lower odds of anemia and iron deficiency than children infected with *P. falciparum* alone. This observation warrants follow-up studies, as there are important implications when implementing integrated control measures against malaria and helminthiases.

## Introduction

Recent estimates indicate that approximately 30% of the world's population is still exposed to malaria and that most clinical events attributable to *Plasmodium falciparum* are concentrated in the African region [1]. Hookworm (*Ancylostoma duodenale* and *Necator americanus*) and other soil-transmitted helminths are also widespread, affecting more than a billion people with an estimated 200 million cases of hookworm infections found in sub-Saharan Africa [2]. Given the widespread distribution of *Plasmodium* and soil-transmitted helminth infections, and the similarity of ecological requirements for disease transmission, coinfection is a common phenomenon. For instance, it has been estimated that over a quarter of school-aged children in sub-Saharan Africa, are at risk of *Plasmodium*-hookworm coinfection [3].

The interactions between soil-transmitted helminths and *P. falciparum*, including immunological responses and clinical outcomes of the host, are not well understood [4]. While some studies observed an inverse relationship between helminthiases and malaria [5]–[8], other studies suggest that coinfection may be more frequent than expected by chance, and hence exacerbate disease of a single infection [9]–[12]. These conflicting patterns have been observed in different age groups for both males and females. Importantly, since chronic helminth infection and *P. falciparum* can lead to anemia, the risk of anemia and iron deficiency among coinfected individuals might be exacerbated [13].

Risk factors and the consequences of single and multiple species parasite infection may vary depending on setting, age, infection intensity, and the host's nutritional status. A deeper understanding of such risk factors has important public health implications, and it is of considerable relevance as control interventions against malaria and helminthiases are going to scale [14], [15].

Here, we report results from a baseline cross-sectional survey as part of a 14-month prospective longitudinal surveillance of anemia in three cohorts (infants aged 6–23 months, children aged 6–8 years, and young women aged 15–25 years) implemented on the site of the recently established Taabo health demographic surveillance system (Taabo HDSS) in south-central Côte d'Ivoire. Emphasis is placed on *P. falciparum* and helminth infections, and micronutrient deficiencies. The specific objectives of the study reported here were (i) to assess the prevalence and intensity of *P. falciparum* and helminth infection, (ii) to evaluate the association between *P. falciparum* and helminth infections in the three population groups, and (iii) to discuss potential implications of *P. falciparum*-hookworm coinfection in relation to anemia in young school-aged children.

## Materials and Methods

### Ethics Statement

Ethical approval was granted by the ethics committee of Basel (EKBB, reference no. 252/09) and Côte d'Ivoire (reference no. 1086 MSHP/CNER). Study investigators were covered by liability insurance (GNA Assurance; Abidjan, Côte d'Ivoire, policy no. 30105811010001). Village authorities and participants were informed about the purpose, procedures, and potential risks and benefits of the study. Written informed consent was obtained from study participants and the parents/guardians of children below the age of 15 years. Suspected clinical malaria (i.e., positive rapid diagnostic test (RDT) and tympanic temperature ≥38°C), severe anemia (i.e., hemoglobin (Hb) <8 g/dl according to national guidelines of Côte d'Ivoire defining anemia requiring appropriate intervention), and helminth infections were treated according to national guidelines.

### Study Area

This study was carried out in the recently established Taabo HDSS, which covers Taabo sub-district located in south-central Côte d'Ivoire [16]. Altitudes of the study area are between 50 and 250 m above sea level. The results presented here stem from the baseline cross-sectional survey of a 14-month prospective longitudinal monitoring, which aims to further the understanding of the etiology of anemia, including measures for prevention and control. The Taabo HDSS consists of a small district town (Taabo Cité), 13 main villages, and over 100 hamlets. Approximately 38,500 people are under demographic and health surveillance. The longitudinal monitoring reported here was implemented in three localities: (i) Taabo Cité, the only small town of Taabo HDSS; (ii) Ahondo, one of the 13 main villages, situated in close proximity to Lake Taabo; and (iii) Katchénou, a hamlet (subsequently designated a small village), located 50 km south of Taabo Cité.

### Field Procedures

Details of the field procedures have been described elsewhere [17]. In brief, we designed a prospective longitudinal study pertaining to anemia and potential nutritional and parasitic risk factors in three age groups, namely (i) infants (aged 6–23 months), (ii) children in early school-age (6–8 years), and (iii) young women (aged 15–25 years). The choice of these three age groups was based on the high vulnerability of infants and women of childbearing age to the consequences of anemia, and the important exposure, yet non-immunity, of young school-aged children to parasitic infections. Based on an estimated proportion of anemia of 60% in infants and school-aged children and 40% in non-pregnant women, a drop-out rate of 20% and a confidence level of 95%, we intended to sample 137 individuals for each of the three age-groups from the readily available Taabo HDSS database, to produce an accurate estimation of the prevalence of anemia with a 9% error margin. Since the participation rate during the initial sampling phase was lower than expected, all individuals meeting our age (and sex) requirements from Ahondo and Katchénou were invited to participate. In Taabo Cité, a stepwise random sampling was employed and 120 infants, 90 children of early school-age, and 90 young non-pregnant women were selected from the Taabo HDSS database.

Overall, 732 individuals were invited to participate. Venous and finger-prick blood, stool, and urine samples were collected and subjected to standardized, quality-controlled methods to diagnose and quantify *Plasmodium* and helminth infections, and determine the participants' micronutrient status.

### Laboratory Analyses

The presence of *P. falciparum* was determined using finger-prick blood and an RDT (ICT ML01 malaria Pf kit; ICT Diagnostics, Cape Town, South Africa). The determination of *Plasmodium* species was done with a thin blood film, and parasitemia assessed with a thick blood film. Hb quantification was done with a portable HemoCue Hb 301 device (HemoCue AB; Ängelholm, Sweden).

Duplicate Kato-Katz thick smears (using 41.7 mg templates) were prepared from each stool sample [18] and examined under a microscope for the presence of soil-transmitted helminth (*Ascaris lumbricoides*, hookworm, and *Trichuris trichiura*) and *Schistosoma mansoni* eggs. The number of eggs was counted and recorded for each species separately. For each individual, the egg counts of both Kato-Katz thick smears were added and multiplied by a factor 12 to obtain a standardized measure of infection intensity (i.e., eggs per gram of stool (EPG)). Urine samples were subjected to a filtration method (10 ml) [19] and slides quantitatively examined under a microscope for *S. haematobium* eggs.

For quality control, 10% of the Kato-Katz thick smears and urine filters were reexamined by a senior technician. In case of conflicting results, the slides were reexamined and results discussed with the technicians until consensus was reached.

Venous blood samples were centrifuged, aliquoted, and kept at −20°C before transfer in an ice-cold box to the Centre Suisse de Recherches Scientifiques en Côte d'Ivoire (Abidjan, Côte d'Ivoire) and then to ETH Zurich (Zurich, Switzerland). Riboflavin was measured by the erythrocyte glutathione reductase activity coefficient (EGRAC) assay, using the method of Dror et al. [20] with some modifications validated in our laboratory. A cut-off value >1.4 was used to define riboflavin deficiency [21]. Serum ferritin, soluble transferrin receptor (sTfR), retinol binding protein (RBP), α1-acid glycoprotein (AGP), and C-reactive protein (CRP) were measured with a sandwich enzyme-linked immunosorbant assay (ELISA) that has been described elsewhere [22].

### Statistical Analysis

Data were entered twice using Microsoft Access version 10.0 (2007 Microsoft Corporation) and the two datasets compared with EpiInfo version 3.4.1 (Centers for Disease Control and Prevention; Atlanta, GA, USA). All statistical analyses were performed with STATA version 10 (StataCorp.; College Station, TX, USA).

Anemia was defined as Hb <11.0 g/dl for infants, <11.5 g/dl for children aged 6–8 years, and <12.0 g/dl for non-pregnant women, according to guidelines put forward by the World Health Organization (WHO) [23]. Storage iron depletion was defined as ferritin <12 µg/l for infants without inflammation, and <15 µg/l for school-aged children and women without inflammation. For participants with AGP >1 g/l or CRP >10 mg/l, storage iron depletion was defined as ferritin <30 µg/l [23]. Cellular iron deficiency was defined as sTfR >8.5 mg/l [24]. Vitamin A deficiency was defined as RBP <0.825 µmol/l [25].

Only those individuals who had complete datasets (i.e., demographic, parasitological, and micronutrient data) were included for detailed statistical analyses (*n* = 324). For calculating household socioeconomic status, an asset-based index was constructed for each household of the participants with complete data records, according to a method described by Filmer and Pritchett [26]. In brief, data on household assets (e.g., radio), housing characteristics (e.g., wall type), and number of people per room were obtained from the Taabo HDSS database. The binary data of these variables were weighted using principal component analysis (PCA), and the households were subsequently divided into five socioeconomic groups (wealth quintiles); namely (i) most poor, (ii) very poor, (iii) poor, (iv) less poor, and (v) least poor. The procedure is further explained and illustrated in technical notes provided by the Health, Nutrition, and Population (HNP) Poverty Thematic Group of the World Bank [27] and elsewhere [28].

Crude odds ratios (ORs) were calculated for variables potentially associated with *P. falciparum* (defined as a positive RDT or the presence of *Plasmodia* on blood films) and hookworm infection. To assess independent predictors of *P. falciparum* and hookworm infection, a multivariate logistic regression model was fitted and standard error adjusted for potential clustering within household. An independent model was computed for each age group.

Student's *t*-test and Wilcoxon rank-sum test were used for comparison of means and ranks, respectively. Categorical data were compared using χ^2^ test or Fisher's exact test, as appropriate.

## Results

### Attrition Analysis

From the 732 individuals invited to participate in our prospective longitudinal study, 407 (55.6%) provided written informed consent and 324 (44.3%) had complete data records (i.e., anthropometric, hematologic, parasitic, and micronutrient data). The sex ratio was balanced among infants and school-aged children who were lost to follow-up and those with complete data. There was no difference in mean age in infants, school-aged children and women who were lost to follow-up and those with complete data. There was a significant difference in participation rate across study settings (Taabo Cité: 44%, Ahondo: 34%, Katchénou: 64%; p<0.001).

### Prevalence of *P. falciparum* Infection and Parasitemia

According to the RDT and the blood film examinations, 58.0% (95% confidence interval (CI) 52.4–63.5%) of the study participants were found infected with *P. falciparum*. Children aged 6–8 years showed the highest prevalence (78.2%). The respective prevalence in infants and non-pregnant women were 45.3% and 36.6% (Table 1). Among those found infected with *P. falciparum*, 30.2% of infants, 9.6% of school-aged children, and 3.3% of young non-pregnant women harbored >5,000 parasites/µl of blood, respectively. Four infants aged between 7 and 14 months and one 8-year-old child presented with clinical malaria.

**Table 1:** Baseline characteristics of soil-transmitted helminth, schistosome, and *P. falciparum* infections in three different age groups.

Characteristics	Infants (*n* = 95)	School-aged children (*n* = 147)	Youn women (*n* = 82)	p-value*
**Soil-transmitted helminth infection**				
Prevalence (total), % (95% CI)	3.2 (0.7–9.0)	29.9 (22.7–38.0)	30.5 (20.8–41.6)	<0.001
Hookworm prevalence, % (95% CI)	1.1 (0.0–5.7)	29.9 (22.7–38.0)	30.5 (20.8–41.6)	<0.001
*Ascaris lumbricoides* prevalence, % (95% CI)	1.1 (0.0–5.7)	2.0 (0.4–5.8)	0	0.695
*Trichuris trichiura* prevalence, % (95% CI)	1.1 (0.0–5.7)	4.1 (1.5–8.7)	1.2 (0.0–6.6)	0.336
Median hookworm egg count (for positive cases), EPG	252	204	72	
Mean hookworm egg count (for positive cases), EPG	252	752	203	
***S. haematobium*** ** infection**				
Prevalence, % (95% CI)	2.1 (0.3–7.4)	10.2 (5.8–16.3)	22.0 (13.6–32.5	<0.001
Median number of eggs of *S. haematobium*/ 10 ml urine (for positive cases)	1	16	11	
Mean number of eggs of *S. haematobium*/ 10 ml urine (for positive cases)	1	38	36	
***P. falciparum*** ** infection**				
Prevalence, % (95% CI)	45.3 (35.0–55.8)	78.2 (70.7–84.6)	36.6 (26.2–48.0)	<0.001
Median number of parasites/µl blood (for positive cases)	2440	740	192	
Mean number of parasites/µl blood (for positive cases)	11461	4163	1081	
**Coinfection**				
Prevalence of *P. falciparum*-hookworm coinfection	1.1 (0.0–5.7)	27.9 (20.8–35.9)	4.9 (1.3–12.0)	<0.001
Prevalence of *P. falciparum*-schistosome coinfection	1.1 (0.0–5.7)	8.8 (4.8–14.6)	8.5 (3.5–16.8)	0.004

Prevalence, median, and arithmetic means, as determined from stool, urine, and finger-prick blood samples collected from 324 individuals in the Taabo health demographic surveillance system in south-central Côte d'Ivoire in April 2010.

* P-value based on χ^2^ or Fisher's exact test, as appropriate, between groups.

CI, confidence interval; EPG, eggs per gram of stool.

### Prevalence and Intensity of Helminth Infection

Overall, 72 of the 324 participants (22.2%) with complete data records had an infection with soil-transmitted helminths. Hookworm was the predominant species (21.6%), whereas only eight (2.5%) and four (1.2%) individuals were infected with *T. trichiura* and *A. lumbricoides*, respectively.

There were only three cases (3.2%) of soil-transmitted helminth infection in infants, whereas a prevalence of 29.9% and 30.5% was found in young school-aged children and non-pregnant women, respectively (Table 1). Among children aged 6–8 years, infection with *A. lumbricoides* and *T. trichiura* was found in three and six individuals, whereas one case of *T. trichiura* was found in non-pregnant women and one case of each *T. trichiura* and *A. lumbricoides* was observed among infants.

*S. haematobium* infection was found in 35 individuals (10.8%) with the highest prevalence observed in non-pregnant women (22.0%). The respective prevalence in school-aged children and infants was 10.2% and 2.1%.

Helminth infections were primarily of low intensity, regardless of the age groups. Only two participants, children aged 6 and 8 years, presented with heavy hookworm infection (≥4000 EPG).

### Parameters Associated with *P. falciparum* and Helminth Infections

Tables 2 and 3 present ORs between demographic, socioeconomic, inflammatory, and micronutrient parameters and *P. falciparum* and hookworm infection, respectively, as determined by univariate and multivariate logistic regression analyses. Our multivariate regression model revealed that vitamin A deficiency (OR = 10.26, 95% CI 1.89–55.54), inflammation (OR = 4.74, 95% CI 1.12–20.04), and setting (OR (Katchénou) = 30.20, 95% CI 2.60–350.65) were significantly associated with *P. falciparum* infection in infants. Vitamin A deficiency (OR = 10.79, 95% CI 2.68–43.49), cellular iron deficiency (OR = 5.38, 95% CI 1.56–18.56), inflammation (OR = 5.36, 95% CI 1.78–16.13), and hookworm infection (OR = 7.47, 95% CI 1.84–30.32) were significantly associated with higher odds of *P. falciparum* infection in children aged 6–8 years. Moreover, the odds of *P. falciparum* infection among school-aged children were significantly lower for older children (OR (8 years) = 0.25, 95% CI 0.07–0.92). For young non-pregnant women, a concurrent hookworm infection was significantly associated with lower odds of *P. falciparum* infection (OR = 0.14, 95% CI 0.03–0.60). Non-pregnant women had a lower odds of *P. falciparum* infection if they belonged to the least poor quintile (OR = 0.06, 95% CI 0.00–0.86).

**Table 2:** Demographic, socioeconomic, parasitological, and micronutrient variables associated with *P. falciparum* infection, stratified by study group.

Variable	Infants (*n* = 95)			School-aged children (*n* = 147)			Young women (*n* = 82)		
	Crude OR	Adjusted OR	*P*	Crude OR	Adjusted OR	*P*	Crude OR	Adjusted OR	*P*
		(95% CI)			(95% CI)			(95% CI)	
**Sex**									
Male	1.00			1.00					
Female	1.08	3.39 (0.71, 16.06)	0.125	0.56	0.53 (0.15, 1.86)	0.128	N/A	–	
**Age class**									
Younger	1.00			1.00			1.00		
Middle	2.10	0.92 (0.18, 4.69)	0.917	1.33	1.04 (0.20, 5.33)	0.961	1.31	0.56 (0.18, 1.75)	0.318
Older	2.33	2.10 (0.48, 9.19)	0.323	0.49	**0.25 (0.07, 0.92)**	**0.036**	N/A	–	
**Setting**									
Taabo Cité	1.00			1.00			1.00		
Ahondo	1.13	1.31 (0.35, 4.85)	0.686	6.17	7.09 (1.02, 49.18)	0.161	1.90	0.52 (0.11, 2.51)	0.417
Katchénou	12.40	**30.20 (2.60, 350.65)**	**0.006**	7.00	3.71 (0.16, 84.87)	0.959	4.46	1.21 (0.11, 12.75)	0.876
**Socioeconomic status**									
Most poor	1.00			1.00			1.00		
Very poor	0.29	0.39 (0.06, 2.54)	0.323	1.40	4.63 (0.54, 39.78)	0.163	0.39	0.20 (0.03, 1.35)	0.099
Poor	0.07	1.12 (0.05, 25.09)	0.943	0.40	2.83 (0.12, 64.27)	0.513	0.18	0.13 (0.01, 1.67)	0.117
Less poor	0.08	1.47 (0.06, 33.61)	0.808	0.41	6.56 (0.25, 172.93)	0.260	0.44	0.36 (0.04, 3.64)	0.385
Least poor	0.04	0.17 (0.01, 5.30)	0.316	0.08	0.37 (0.01, 10.56)	0.560	**0.10**	**0.06 (0.00, 0.86)**	**0.039**
**Hookworm infection**	N/A	-		**5.36**	**7.47 (1.84, 30.32)**	**0.005**	**0.23**	**0.14 (0.03, 0.60)**	**0.009**
**Schistosome infection**	N/A	-		2.08	0.57 (0.06, 5.71)	0.632	1.01	2.42 (0.59, 9.94 )	0.222
**Cellular iron deficiency**	2.52	4.15 (0.93, 18.46)	0.061	2.66	**5.38 (1.56, 18.56)**	**0.008**	1.13	2.20 (0.50, 9.73)	0.299
**Riboflavin deficiency**	1.08	0.65 (0.21, 2.00)	0.451	0.61	0.59 (0.14, 2.52)	0.474	0.61	0.70 (0.22, 2.23)	0.551
**Vitamin A deficiency**	3.90	**10.26 (1.89, 55.54)**	**0.007**	4.03	**10.79 (2.68, 43.49)**	**0.001**	N/A	–	
**Inflammation (AGP)**	7.59	**4.74 (1.12, 20.04)**	**0.034**	3.10	**5.36 (1.78, 16.13)**	**0.003**	3.27	2.70 (0.22, 2.54)	0.642

In April 2010, stool, urine, and blood samples were collected from 324 individuals and socio-demographic variables obtained from the readily available database of the Taabo health and demographic surveillance system located in south-central Côte d'Ivoire. Univariate and multivariate logistic regressions were used to calculate the association between *P. falciparum* infection as outcome and demographic, geographic, socioeconomic, parasitic, micronutrient, and inflammatory explanatory variables. Adjusted odds ratios (OR) are reported with their 95% confidence intervals (CI). Significant associations are in bold.

N/A, not applicable.

**Table 3:** Demographic, socioeconomic, parasitological, and micronutrient variables associated with hookworm infection, stratified by school-aged children and women.

Variable	School-aged children (*n* = 147)			Women (*n* = 82)		
	Crude OR	Adjusted OR	*P*	Crude OR	Adjusted OR	*P*
		(95% CI)			(95% CI)	
**Sex**						
Male	1.00					
Female	0.53	0.50 (0.17, 1.48)	0.209	N/A	–	
**Age class**						
Younger	1.00			1.00		
Middle	0.82	0.87 (0.18, 4.09)	0.859	0.77	0.87 (0.26, 2.87)	0.728
Older	2.12	**4.56 (1.11, 18.69)**	**0.035**	N/A	–	
**Setting**						
Taabo Cité	1.00			1.00		
Ahondo	5.34	4.82 (0.80, 28.97)	0.086	0.84	0.28 (0.04, 1.94)	0.200
Katchénou	43.43	**7.85 (1.05, 58.82)**	**0.045**	0.92	0.99 (0.08, 12.29)	0.991
**Socioeconomic status**						
Most poor	1.00			1.00		
Very poor	0.23	0.23 (0.04, 1.23)	0.085	0.54	0.48 (0.07, 3.12)	0.443
Poor	0.07	**0.07 (0.01, 0.84)**	**0.036**	2.53	2.88 (0.23, 36.46)	0.414
Less poor	0.02	**0.02 (0.00, 0.24)**	**0.003**	0.59	0.64 (0.05, 8.65)	0.735
Least poor	0.13	0.13 (0.01, 2.36)	0.168	0.87	0.41 (0.02, 8.28)	0.564
***P. falciparum*** ** infection**	5.36	**7.29 (1.36, 39.23)**	**0.021**	0.23	**0.16 (0.05, 0.55)**	**0.004**
**Schistosome infection**	1.07	1.39 (0.33, 5.85)	0.655	1.97	2.58 (0.63, 10.50)	0.187
**Iron deficiency (sTfR)**	0.46	0.32 (0.10, 1.03)	0.055	0.78	0.80 (0.23, 2.85)	0.735
**Riboflavin deficiency**	1.24	2.42 (0.71, 8.25)	0.157	0.59	0.49 (0.16, 1.44)	0.194
**Vitamin A deficiency**	2.47	1.42 (0.37, 5.41)	0.612	N/A	–	
**Inflammation (AGP)**	0.74	0.90 (0.27, 2.96)	0.863	0.74	1.48 (0.26, 8.37)	0.657

In April 2010, stool, urine and blood samples were collected from 324 individuals and socio-demographic variables obtained from the readily available database of the Taabo health and demographic surveillance system located in south-central Côte d'Ivoire. Univariate and multivariate logistic regressions were used to calculate the association between hookworm infection as outcome and demographic, geographic, socioeconomic, parasitic, micronutrient, and inflammatory explanatory variables. Adjusted odds ratios (OR) are reported with their 95% confidence intervals (CI).. Significant associations are in bold.

N/A, not applicable.

Following multivariate analysis with hookworm infection as outcome, age 8 years (OR = 4.56, 95% CI 1.11–18.69), infection with *P. falciparum* (OR = 7.29, 95% CI 1.36–39.23), and setting (OR (Katchénou) = 7.85, 95% CI 1.05–58.82) were significantly positively associated with hookworm infection in school-aged children. Intermediate socioeconomic status was associated with decreased odds of hookworm infection among these children. Furthermore, non-pregnant women infected with *P. falciparum* had lower odds of hookworm infection (OR = 0.16, 95% CI 0.05–0.55). Multivariate logistic regression showed that setting (Ahondo) was the only variable significantly associated with schistosome infection in children and women.

### Prevalence and Implications of *P. falciparum*-Hookworm Coinfection

As shown in Table 1, *P. falciparum*-helminth coinfection was most prevalent among children aged 6–8 years. The prevalence of a concurrent infection with *Plasmodium* and hookworm was 27.9% in school-aged children, whilst the respective prevalence in young non-pregnant women and infants was 4.9% and 1.1%. The prevalence of *P. falciparum*-*Schistosoma* coinfection was low in all age groups.

Considering the overall low prevalence of *P. falciparum-Schistosoma* coinfection and *P. falciparum*-hookworm coinfection in infants and women, further investigations focused on *P. falciparum*-hookworm coinfection in the school-aged children. Multivariate regression analysis revealed that sex (OR (female) = 0.33, 95% CI 0.12–0.94) and setting (OR (Ahondo) = 7.53, 95% CI 1.22–46.52; OR (Katchénou) = 11.98, 95% CI 1.64–87.20), was significantly associated with *P. falciparum*-hookworm coinfection among school-aged children (compared to no infection or single species infection). Other demographic, parasitic, and micronutrient parameters were not significantly associated with coinfection status.

Children coinfected with hookworm and *P. falciparum* had significantly higher concentrations of Hb (Wilcoxon's rank-sum test, p = 0.038 (*n* = 115) and lower sTfR concentrations (Wilcoxon's rank-sum test, p = 0.010 (*n* = 115)) than children infected with *P. falciparum* alone. Adjusting for socioeconomic status, sex, age, *P. falciparum* parasitemia, stunting, inflammation status, and setting revealed that children with a coinfection had significantly lower odds of cellular iron deficiency (OR = 0.17, 95% 0.04–0.70) and anemia (OR = 0.23, 95% CI 0.06–0.83) than their counterparts harboring a single infection with *P. falciparum*. There was an interaction between age and infection status. Table 4 shows the hematological and inflammation data for school-aged children, stratified by 1-year age increments. Children aged 8 years with *P. falciparum*-hookworm coinfection had significantly higher Hb concentration and lower median values of sTfR, sTfR/log ferritin ratio, and AGP than those infected with *P. falciparum* alone. There was a similar trend for Hb, sTfR, and sTfR/log ferritin in children aged 7 years.

**Table 4:** Implications of *P. falciparum*-hookworm coinfection among school-aged children, stratified by age.

Variable	6-year-old (*n* = 45)			7-year-old (*n* = 43)			8-year-old (*n* = 59)		
	*P. falciparum* infection	*P. falciparum-*hookworm coinfection	*P*	*P. falciparum* infection	*P. falciparum-*hookworm coinfection	*P*	*P. falciparum* infection	*P. falciparum-*hookworm coinfection	*P*
Mean hemoglobin, g/dl (standard deviation)	11.3 (1.0)	11.0 (1.5)	0.739	11.4 (1.0)	11.9 (1.0)	0.182	11.0 (1.1)	11.9 (1.0)	0.009
Geometric mean *P. falciparum* parasitemia (95% CI)	1,141.3 (495.6, 2,626.4)	138.6 ( 23.2, 840.7)	0.013	490.9 (214.4, 1,122.1)	678.8 (227.0, 2,017.3)	0.915	280.5 (61.2, 1,272.7)	825.5 (480.5, 1,417.8)	0.457
Median sTfR, mg/l (interquartile range)	8.1 (6.7, 9.5)	8.7 (6.9, 10.9)	0.987	9.0 (7.0, 11.4)	6.7 (5.8, 9.7)	0.107	9.2 (8.0, 11.0)	7.6 (6.5, 8.3)	0.007
Median serum ferritin, µg/l (interquartile range)	75.0 (57.2, 140.1)	62.9 (55.1, 85.8)	0.485	61.1 (39.5, 108.2)	73.1 (63.3, 94.0)	0.229	62.2 (53.7, 116.3)	66.8 (54.1, 81.5)	0.715
Median sTfR/log ferritin (interquartile range)	4.4 (3.4, 5.1)	4.6 (3.8, 5.0)	0.790	5.1 (3.6, 6.4)	4.0 (3.1, 4.6)	0.047	5.0 (4.3, 5.8)	3.9 (3.5, 5.1)	0.035
Median AGP, g/l (interquartile range)	1.0 (0.8, 1.2)	1.0 (0.7, 1.1)	0.361	0.9 (0.7, 1.0)	0.9 (0.9, 1.0)	0.366	1.0 (0.9, 1.1)	0.8 (0.7, 1.0)	0.020
Median CRP, mg/l (interquartile range)	3.2 (0.8, 13.6)	1.2 (0.6, 2.2)	0.104	1.4 (0.6, 3.4)	2.6 (0.8, 5.7)	0.436	1.8 (0.9, 8.1)	3.3 (0.5, 4.2)	0.794

Arithmetic, geometric, and median values were derived from blood samples collected from 147 school-aged children in the Taabo health demographic surveillance system in south-central Côte d'Ivoire in April 2010. Children infected with *P. falciparum* alone are compared with children matched for age coinfected with *P. falciparum* and hookworm. Unpaired bilateral *t*-test was performed to compare means of hemoglobin concentration between groups. Wilcoxon rank-sum test was applied to compare *P. falciparum* parasitemia, sTfR, serum ferritin, ratio sTfR/log ferritin, AGP, and CRP values between groups.

AGP, α1-acid glycoprotein; CI, confidence interval; CRP, C-reactive protein; sTfR, soluble transferrin receptor.

## Discussion

Our data derived from a baseline cross-sectional survey among three age groups in south-central Côte d'Ivoire confirm that *P. falciparum* and helminths co-exist with school-aged children at highest risk of coinfection [29], [30]. We found significant associations between infection status and demographic parameter (age), social-ecological systems (setting and socioeconomic status), and host inflammatory and micronutrient status. Interestingly, young non-pregnant women infected with *P. falciparum* showed significantly lower odds of concurrent hookworm infection, whilst there was a significant positive association between both infections in children aged 6–8 years. Children coinfected with *P. falciparum* and hookworm had lower odds of anemia and cellular iron deficiency than their counterparts infected with *P. falciparum* alone. Our findings therefore underscore that the interactions between *P. falciparum* and hookworm are complex and may benefit the host in some circumstances.

### Demographic Variables

The finding that, in school-aged children, boys had a significantly higher odds of coinfection might reflect different recreational exposures between genders. Hence, we cannot generalize our findings about parasites interactions in young women (studied here) to young men (not studied) as the two groups might be exposed differently to parasitic infection [31]. In school-aged children, growing older was associated with lower odds of *P. falciparum* infection, and the infant-group showed highest *P. falciparum* parasitemia. Moreover, whilst *P. falciparum* infection was associated with inflammation in infants and school-aged children, there was no significant relationship between both variables in young non-pregnant women. These observations most likely reflect the clinical and parasitological semi-immunity that individuals living in malaria-endemic areas built up and which protect them from high parasitemia, clinical malaria, and facilitates the clearance of *Plasmodium* [32]. The observation that children aged 8 years had higher odds of hookworm infection compared with their 6-year-old counterparts might be explained by a longer probability of being infected with parasitic worms. Highest helminth infection prevalence is indeed often observed among older children, adolescents, and young adults [33].

### Social-Ecological Parameters

Our findings that children at early school-age who live in remote rural areas (i.e., in this study, Katchénou) are at higher risk of hookworm infection and *P. falciparum*-hookworm coinfection might be explained by the particularly poor hygiene status and suitable ecological conditions for the parasites life-cycles as well as a difficult access to health care facilities for individuals living in this setting. Indeed, at the time of our study, none of the households in Katchénou had sanitation and all of them obtained drinking water from a community pump. The nearest health center was situated in Sokrogbo, a village located approximately 5 km from Katchénou with difficult access particularly during the rainy season. The observation that remote rural setting (i.e., Katchénou) was significantly associated with *P. falciparum* infection in infants but not in school-aged children might be explained by the high malaria transmission in this setting, increasing the probability that young infants become infected. The high prevalence of *P. falciparum* in all the three study groups emphasizes the importance of implementing prevention and control measures against malaria [34]. Among infants aged 6–23 months, 45% were infected with *P. falciparum* and the parasitemia level in this age group was higher than in children aged 6–8 years and in non-pregnant women aged 15–25 years. Hence, emphasis should be placed on protecting infants from mosquito bites and on effective management of malaria cases during childhood, most importantly in remote areas where prompt access to quality health care remains a formidable challenge.

### Micronutrient Status

With regard to micronutrients, it is not surprising that vitamin A deficiency appeared to be associated with *P. falciparum* infection in infants and school-aged children. Indeed, we have previously shown that *P. falciparum* infection and vitamin A deficiency are prevalent among infants and school-aged children living in our study area [17]. Furthermore, although WHO does not recommend vitamin A supplementation before 6 months of age [35]–[37], several studies have shown the role and the importance of vitamin A in the pathology of malaria [38], [39] and, more generally, in the development of the immunological system during childhood [40]–[42]. It has also been observed that vitamin A utilization may increase during a clinical malaria episode [43], [44], suggesting a vicious cycle between deficiency in vitamin A and *Plasmodium* infection. The cross-sectional nature of our study, however, does not allow drawing causal inference. There are several hypotheses for the significant association we found between iron deficiency and *P. falciparum* infection in infants, based on the hematological and inflammatory consequences of *Plasmodium* infection. On the one hand, erythropoiesis rate might indirectly be increased in response to *Plasmodium* infection, to compensate for the loss of erythrocytes through hemolysis and splenic clearance of infected and uninfected erythrocytes. This increased erythropoiesis might, in turn, be translated to increased sTfR concentrations [45], [46]. On the other hand, the effect of chronic or acute inflammation on sTfR concentrations is still being debated. Whilst several studies have suggested that inflammation inhibits erythropoiesis, others have reported higher sTfR concentrations in individuals with inflammation [47], [48]. This latest observation might be explained by a secondary hematological response to hepcidin-mediated iron sequestration during inflammatory states.

### Interactions and Potential Implications of *P. falciparum*-Hookworm Coinfection

Interestingly, our results suggest that the negative association observed between *P. falciparum* and hookworm infection in young non-pregnant women was independent from the social-ecological context. Indeed, multivariate regression analysis showed that this association was independent from age, socioeconomic status, and type of setting of the participating women. We did not find any significant relationship between Hb concentrations and hookworm infection intensity, but it should be kept in mind that our survey was conducted in a setting where helminth infection were primarily of light intensity, probably preventing an important intestinal blood loss in infected subjects [49]. One hypothesis for the observed negative association between *P. falciparum* and hookworm infection in women is based on the distinct immunological regulations stimulated by the two parasitic infections [4]. Indeed, helminths are known to activate the immune system with a strong polarization toward T helper 2 (Th2) responses. Furthermore, helminths are often able to survive many years in the host through the induction of immmunoregulatory mechanism resulting in an anti-inflammatory environment [50], [51]. The immune responses to *Plasmodium* are more complex and depend on the species and the stage of infection. The host response toward early infection is rather based on Th1 activation and the production of pro-inflammatory cytokines [52]. These non-specific reactions switch in later stage of *Plasmodium* infection to a Th2 cytokines profile, leading to the production of specific antibodies [53]. Hence, one may suggest that women infected with one parasite species have a more efficient immunological system than women free of infection, leading to a more rapid clearance of a concurrent parasitic infection. Of note, many studies have reported a beneficial effect of helminth infection on malaria [5], [6], [54]. An alternative hypothesis is that a specific parasitic infection may deprive a concurrent infection of iron or another nutrient necessary for parasite growth, leading to the elimination of the latter acquired infection. In our cohort, however, women infected with hookworm or *P. falciparum* singly, did not have lower concentrations of serum ferritin or sTfR than their non-infected counterparts. The observation that children aged 6–8 years coinfected with *P. falciparum* and hookworm had lower odds of cellular iron deficiency and anemia, compared to children infected with *P. falciparum* alone, adds to the current debate about *Plasmodium*-helminth coinfection outcomes. A protective effect on anemia has been observed in early school-aged children concurrently infected by *P. vivax* and soil-transmitted helminths [8]. The observation that 7- and 8-year-old children infected with *P. falciparum* alone had lower Hb and higher sTfR concentrations compared with children coinfected with hookworm, whilst serum ferritin concentrations were normal in both groups, suggests that mono-infected children might be more prone to anemia due to tissue iron deficiency, potentially associated with inflammation [47]. Of note, AGP and *P. falciparum* parasitemia were significantly correlated in children infected with *P. falciparum* alone (*n* = 67; Spearman's ρ: 0.27; p = 0.025) and this association was not significant for children coinfected with hookworm (*n* = 39; Spearman's ρ: 0.04; p = 0.807). Moreover, AGP concentrations were significantly higher in 8-year-old coinfected children than in children matched for age infected with *P. falciparum* only, indicating that inflammation might indeed be less important in coinfected children as compared with children infected with *P. falciparum* singly. The measure of hepcidin and inflammatory cytokines and implementation of an anthelmintic drug intervention trial would shed new light on this hypothesis.

In conclusion, our findings emphasize that *P. falciparum* and hookworm infections are associated with demographic, social-ecological, and host inflammatory and micronutrient factors. Coinfection outcomes are complex and might depend on the age and the immune system of the host. New research is needed both in the laboratory and in the field to deepen our understanding of the mechanisms and public health implications of *P. falciparum*-hookworm coinfection. Our observations that coinfection with *P. falciparum* and hookworm are particularly prone in a specific age group (i.e., school-aged children) calls for concerted action in this group. Finally, the finding that light hookworm infection may prevent anemia in children coinfected with *P. falciparum* should be considered when implementing integrated prevention and control measures targeting helminthiases and malaria, and call for a surveillance-response approach, so that specific interventions do not harm existing adaptive immune defense mechanisms which might exacerbate morbidity.
